# Usefulness of the synthesized 18‐lead ECG in premature ventricular contractions from near the HIS bundle

**DOI:** 10.1002/joa3.12834

**Published:** 2023-02-20

**Authors:** Hirofumi Matsumoto, Hirota Kida, Rieko Nakanishi, Nobuyuki Ogasawara

**Affiliations:** ^1^ Department of Clinical Engineering Japan Community Health care Organization Osaka Hospital Osaka Japan; ^2^ Department of Clinical Engineering Osaka General Medical Center Osaka Japan; ^3^ Department of Clinical Engineering Nara Prefectural Seiwa Medical Center Nara Japan; ^4^ Department of Cardiology Japan Community Healthcare Organization Osaka Hospital Osaka Japan

**Keywords:** ablation, near the HIS bundle, premature ventricular contractions, synthesized 18‐lead ECG

Isolated premature ventricular complexes (PVCs) are the most common arrhythmias observed in patients even if without structural heart disease.^1^ Although most PVCs originate from the right ventricular outflow tract (RVOT), there are some PVCs that originate near the HIS bundle.^2^ The synthesized 18‐lead ECG consists of right‐sided thoracic (V3R, V4R, V5R) and dorsal (V7, V8, V9) ECG leads derived from a mathematical calculation of the 12‐lead ECG, and the detailed mathematical formula is shown in Figure 1. The synthesized 18‐lead ECG has been reported to be effective for PVCs of an RVOT origin, but the rest remains to be elucidated.^3^ The aim of this study was to investigate the characteristics of the synthesized 18‐lead ECG in patients with PVCs originating from near the HIS bundle.

**FIGURE 1:**
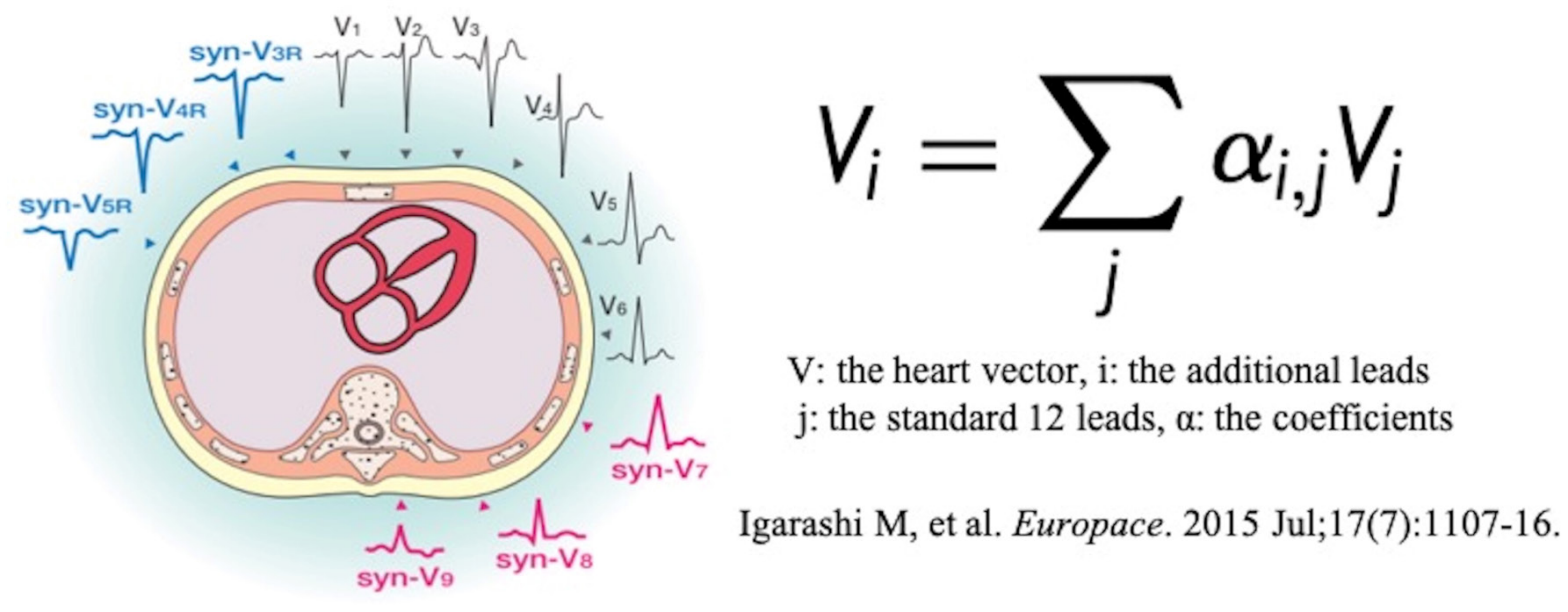
Schematic of the synthesized 18‐lead ECG. The 18‐lead ECG is synthesized from the 12‐lead ECG by a specific arithmetic process.

The synthesized 18‐lead ECGs of 13 consecutive patients who underwent radiofrequency catheter ablation (RFCA) of idiopathic PVCs from near the HIS bundle at the Japan Community Healthcare Organization Osaka Hospital, Osaka General Medical Center, and Nara Prefectural Seiwa Medical Center from January 2018 to December 2020 were included in this retrospective analysis. The origin of the PVCs was identified by a cardiologist using a 3D mapping system (CARTO 3: Biosense Webster Inc or EnSite: Abbott Medical Inc). We analyzed the QRS morphology pattern of the synthesized 18‐lead ECG analyzed in those patients. This study was approved by the Ethics Committee of each hospital in accordance with the Declaration of Helsinki.

The synthesized 18‐lead ECG and ECG characteristics in 13 patients with PVCs from near the HIS bundle are shown in Figure 2 and Table 1. The mean QRS duration of the ECG was 142.5 ± 26.9 ms. In 12 of 13 patients, a left bundle branch block pattern (92.3%) was present, and 11 of 13 patients had inferior axis deviation (84.6%). Regarding the right‐sided chest leads of the synthesized 18‐lead ECG, in all patients, a QS pattern in lead V5R (100%) was observed, and in 12 of 13 patients, a QS pattern in leads V3R, V4R, and V5R (92.3%) was present. A comparison showed that the synthesized ECGs recorded during sinus rhythm and during PVCs were not consistent, as shown in Table 2.

**FIGURE 2:**
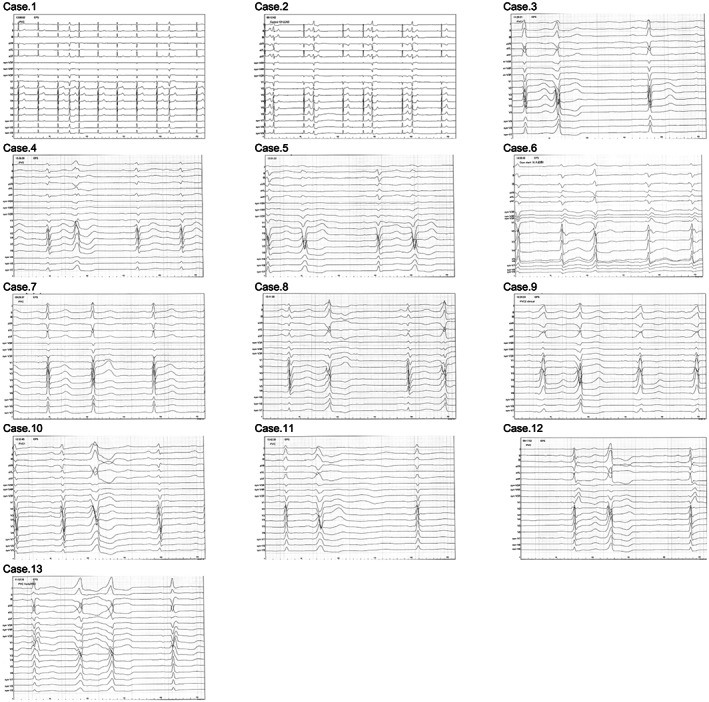
Details of the ECGs of the 13 cases. Only the 7th case is considered to be a PVC caused by the HIS bundle based on the characteristics of the ECG.

**TABLE 1:** ECGs findings and characteristics of 13 cases.

No	Gender	Age	Transitional zone	Axis	Pattern	Duration (ms)	Right‐sided chest leads				Back leads		
							I	V5R	V4R	V3R	V7	V8	V9
1	M	71	V3	Inferior	LBBB	170	R	QS	QS	QS	R	R	R
2	M	76	V3	Inferior	LBBB	142	R	QS	QS	QS	R	R	R
3	M	77	V3.5	Inferior	LBBB	148	r	QS	QS	QS	R	R	R
4	M	87	V1.5	Superior	RBBB	140	r	QS	QS	QS	R	R	R
5	M	78	V4	Superior	LBBB	144	r	QS	QS	QS	R	R	R
6	M	80	V2.5	Inferior	LBBB	122	r	QS	QS	QS	R	R	R
7	M	51	V3.5	Inferior	LBBB	70	r	QS	QS	QS	R	R	R
8	F	65	V2.5	Inferior	LBBB	136	r	QS	QS	QS	R	R	R
9	M	80	V3.5	Inferior	LBBB	144	r	QS	QS	QS	R	R	R
10	M	45	V3	Inferior	LBBB	176	r	QS	QS	QS	R	R	R
11	F	70	V3	Inferior	LBBB	168	r	QS	QS	QS	R	R	R
12	F	64	V3	Inferior	LBBB	160	r	QS	rS	rS	R	R	R
13	M	67	V2	Inferior	LBBB	132	r	QS	QS	QS	R	R	R

Abbreviations: ECG, electrocardiogram; LBBB, left bundle branch block.

**TABLE 2:** Comparison of the synthesized ECG during sinus rhythm and during a PVC originating from near the HIS bundle.

No	Sinus							PVC						
	Duration (ms)	Right‐sided chest leads			Back leads			Duration (ms)	Right‐sided chest leads			Back leads		
		V5R	V4R	V3R	V7	V8	V9		V5R	V4R	V3R	V7	V8	V9
1	71	QS	rS	rS	qRS	qRS	qRS	170	QS	QS	QS	R	R	R
2	67	R	rS	rS	qRS	qRS	qRS	142	QS	QS	QS	R	R	R
3	65	QS	QS	QS	qRS	qRS	qRS	148	QS	QS	QS	R	R	R
4	74	QS	QS	rS	qRS	rS	rS	140	QS	QS	QS	R	R	R
5	65	rS	QS	rS	qRS	rS	rS	144	QS	QS	QS	R	R	R
6	73	rS	rS	rS	rS	rS	rS	122	QS	QS	QS	R	R	R
7	72	rS	rS	rS	qRS	qRS	qRS	70	QS	QS	QS	R	R	R
8	70	rS	rS	rS	R	R	R	136	QS	QS	QS	R	R	R
9	66	R	R	R	qR	qR	qR	144	QS	QS	QS	R	R	R
10	68	QS	QS	QS	R	R	R	176	QS	QS	QS	R	R	R
11	70	QS	rS	rS	qR	qR	qR	168	QS	QS	QS	R	R	R
12	72	R	qR	qR	R	R	R	160	QS	rS	rS	R	R	R
13	76	QS	QS	rS	R	qRS	qRS	132	QS	QS	QS	R	R	R

Abbreviation: ECG, electrocardiogram.

Several studies have shown that identifying PVCs originating from the vicinity of the ventricular septum remains complicated,^4^ and it is difficult to identify the origin of PVCs originating from near the HIS bundle based on the characteristics of the 12‐lead ECG. Noteworthy among our results was that there was a QS pattern in lead V5R in 100.0%. Igarashi et al reported that in the synthesized 18‐lead ECG recorded by pace mapping in the outflow tract, a QS pattern in lead V5R was observed in about 80% at the right coronary cusp (RCC), about 20% in the posterior RVOT, and nothing at the other sites.^3^ Combining the above study^3^ with our results, a QS pattern in lead V5R might have been specific to PVCs originating from near the HIS bundle and RCC. QRS morphology changes after RFCA are often observed in patients with idiopathic PVCs.^5^ In such cases, the use of the synthesized 18‐lead ECG's right‐sided chest leads may assist in the re‐mapping process. Regarding the mechanism of the QS pattern in lead V5R, in general the HIS bundle is anatomically closer to the RCC, and electrical conduction may propagate with a vector away from the right‐sided chest leads such as lead V5R. Based on the characteristics of the ECG, only case 7 was considered to have a PVC from the HIS bundle. An identification of PVCs originating from near the HIS bundle or from the HIS bundle was attempted based on the characteristics of the synthesized ECG, but was not possible due to an insufficient number of cases. Although a 3D mapping system was used in this study, the distance between the HIS bundle and the ablation site was not measured, and it was considered necessary to include the distance in future studies.

A QS pattern recorded in lead V5R of the synthesized 18‐lead ECG was characteristic of PVCs originating from near the HIS bundle. This observation might assist in identifying the origin of PVCs during RFCA.

## AUTHOR CONTRIBUTIONS

H Kida, R Nakanishi, and N Ogasawara participated in the design of the study and coordination and helped draft the manuscript. All authors read and approved the final manuscript.

## CONFLICT OF INTEREST STATEMENT

All authors declare no conflicts of interest associated with this manuscript.

## ETHICS APPROVAL

This study was approved by the Ethics Committee of Japan Community Healthcare Organization Osaka Hospital, Osaka General Medical Center, and Nara Prefectural Seiwa Medical Center in accordance with the Declaration of Helsinki.

## PATIENT CONSENT STATEMENT

Since this study was a retrospective study, we used the opt‐out system according to the ethics guidelines on human medical research by the Japanese government.
